# New long-proboscid lacewings of the mid-Cretaceous provide insights into ancient plant-pollinator interactions

**DOI:** 10.1038/srep25382

**Published:** 2016-05-05

**Authors:** Xiu-Mei Lu, Wei-Wei Zhang, Xing-Yue Liu

**Affiliations:** 1 Department of Entomology, China Agricultural University, Beijing 100193, China; 2 Three Gorges Entomological Museum, P.O. Box 4680, Chongqing 400015, China

## Abstract

Many insects with long-proboscid mouthparts are among the pollinators of seed plants. Several cases of the long-proboscid pollination mode are known between fossil insects (e.g., true flies, scorpionflies, and lacewings) and various extinct gymnosperm lineages, beginning in the Early Permian and increasing during the Middle Jurassic to Early Cretaceous. However, details on the morphology of lacewing proboscides and the relevant pollination habit are largely lacking. Here we report on three lacewing species that belong to two new genera and a described genus from mid-Cretaceous (Albian-Cenomanian) amber of Myanmar. All these species possess relatively long proboscides, which are considered to be modified from maxillary and labial elements, probably functioning as a temporary siphon for feeding on nectar. Remarkably, these proboscides range from 0.4–1.0 mm in length and are attributed to the most diminutive ones among the contemporary long-proboscid insect pollinators. Further, they clearly differ from other long-proboscid lacewings which have a much longer siphon. The phylogenetic analysis indicates that these Burmese long-proboscid lacewings belong to the superfamily Psychopsoidea but cannot be placed into any known family. The present findings represent the first description of the mouthparts of long-proboscid lacewings preserved in amber and highlight the evolutionary diversification of the ancient plant-pollinator interactions.

The order Neuroptera (lacewings), which is an early outshoot of holometabolans, represents a significant lineage in the evolution of insects. Adult lacewings are generally delicate insects in that they possess a soft body and two pairs of membranous wings with highly reticulate venation. Remarkably, lacewing larvae, which are characterized by specialized sucking-mandibulate mouthparts, show spectacularly disparate morphological diversity and life history, as evidenced by termitophile predators (Berothidae), cuckoo-style predators in hymenopteran nests or spider egg cases (Mantispidae), arboreal predators (e.g. Chrysopidae, Hemerobiidae and Coniopterygidae), ambush predators in the soil (e.g. Nemopteridae and Myrmeleontidae), and subterranean herbivores^1^,^2^,^3^. The antiquity of Neuroptera and their relictual nature are considered the reasons why the group today exhibits a rich diversity of radically divergent morphologies and highly specialized life histories^4^,^5^. Hitherto, the extant Neuroptera comprise 16 families in ca. 6000 species^6^, while the fossil record for lacewings is extremely rich and includes many extinct families that existed during the Permian and throughout the Mesozoic^7^. The most dramatic diversity of lacewings occurred in the Jurassic and Cretaceous palaeofauna and gave rise many morphologically specialized groups, such as the giant butterfly-like Kalligrammatidae, the pinnate leaf-mimic Saucrosmylidae, and the minute two-winged mantispid-like Dipteromantispidae^8^,^9^,^10^.

Generally, adults of lacewings are predators. Nonetheless, there are minor exceptions, e.g., some species of Osmylidae, Sisyridae (spongillaflies), Chrysopidae (green lacewings), Myrmeleontidae (ant lions), and Nemopteridae (spoon-winged or thread-winged lacewings), feed on plant material, such as bark flakes, pollen, and honeydew. Among them is a species of *Nemoptera* (Nemopteridae) with relatively specialized mouthparts that are basically mandibulate with slenderly elongated parts of maxillae and labium; the morphological relevance of the proboscis with respect to pollen-feeding has been documented in detail^11^. Interestingly, recently discovered species of an extinct lineage of butterfly-like Middle Mesozoic Kalligrammatidae were associated with a peculiar pollination mode due to the siphonate mouthparts that range from a slender proboscis of 11 mm to robust proboscides of 20–25 mm length^12^.

Kalligrammatidae was considered to belong to the neuropteran clade Psychopsoidea, which currently also includes Psychopsidae, Osmylopsychopidae, and Aetheogrammatidae^10^. Psychopsoids are characterized by broad wings mostly with dilated costal space, branched recurrent veinlet, and richly branched veins^13^, but an unambiguous autapomorphy of this clade has not yet been established. Psychopsidae (silky lacewings) is the only extant family of Psychopsoidea and has been placed together with Nemopteridae, Nymphidae, Myrmeleontidae and Ascalaphidae in the well-defined monophyletic assemblage Myrmeleontiformia, and today Psychopsidae comprises six genera and 27 species distributed in southern Africa, southeastern Asia and Australia^6^. In addition, there are approximately 25 genera and 40 species of fossil psychopsids known from the Triassic to Tertiary^13^,^14^. Psychopsidae is regarded to be closely related to the extinct family Osmylopsychopidae which was a dominant psychopsoid group during the Mesozoic^13^. However, the taxonomy of fossil psychopsids is in great need of revision since many genera and species have uncertain familial positions due to poorly preserved, fragmentary or incomplete wings in fossils^13^.

The natural history of Psychopsidae is poorly known for the majority of species^15^. The most intriguing biological trait of adult silky lacewings is the in-flight ovipositional behavior^15^, but there are no records on their feeding habits. The larvae of silky lacewings are carnivorous and are reported to feed on small arthropods living under tree bark^16^. The biology of other psychopsoid families, i.e. Osmylopsychopidae and Aetheogrammatidae, are completely unknown.

Here we report on three lacewing species based on exquisitely preserved specimens from mid-Cretaceous amber of Myanmar. These species are considered to belong to the superfamily Psychopsoidea based on various wing features. However, they greatly differ from each other in details of the wing venation, and cannot be assigned to any of the known families of Psychopsoidea. Strikingly, all these species possess highly specialized mouthparts with remarkably elongated elements. We compared the mouthpart morphology of these species with contemporary kalligrammatids that have siphonate mouthparts and found differences in detail that are possibly related to divergent host plants. These new fossil discoveries provide significant evidence for understanding the insect-plant interactions during mid-Cretaceous when angiosperms diversified.

## Systematic palaeontology

**Order Neuroptera Linnaeus, 1758**

**Superfamily Psychopsoidea Handlirsch, 1906**

**Family Incertae sedis**

**Figure 1:**
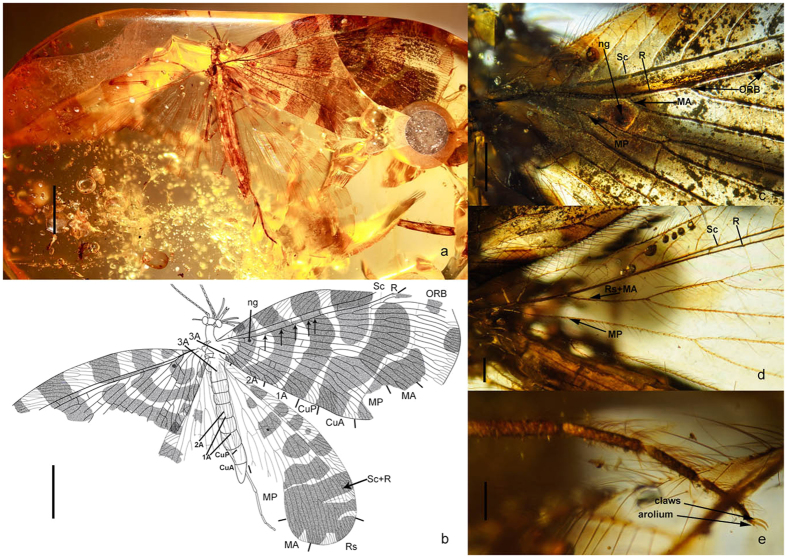
*Fiaponeura penghiani* gen. et sp. nov. (**a**) habitus photo, dorsal; (**b**) habitus drawing; (**c**) photo of proximal part of forewing; (**d**) photo of proximal part of hindwing; (**e**) photo of foreleg tarsus. Scale bar: 2 mm (**a**); 2.5 mm (**b**); 0.5 mm (**c**); 0.2 mm (**d**); 0.05 mm (**e**).

**Genus** ***Fiaponeura*** **gen. nov.** (Figure 1)

**Type species:** *Fiaponeura penghiani* sp. nov.

### Diagnosis

Medium-sized lacewings (forewing length ~11.59 mm). Head (Fig. 1) orthognathous, feebly domed dorsally. Compound eye with maximum diameter nearly equal in length to distance between inner margins of eyes. Ocelli absent. Antenna filiform, densely hairy, at least longer than length of head plus prothorax. Mouthparts composed of a bifid labrum, reduced mandibles, and conspicuously elongated maxillae and labium; maxilla with a long blade-like lobe putatively composed of galea or galea +lacinia, ~2/3 the length of maxillary palp; labium with elongated, distally bifid ligula, which is ~2/3 the length of labial palp. Pronotum about twice as long as wide, slightly narrower than head. Legs slender, each leg with a tibial spur. Wings broad, densely veined, with transversely band-like dark markings. Forewing subtriangular, with round distal margin; trichosores present on almost entire wing margin; a proximal nygma present; costal space narrow at base but distinctly broadened at middle, with a short simple humeral veinlet slightly bent to wing base, and with simple and forked costal crossveins, among which no interlink veinlet is present; subcostal space ~1/4 as wide as costal space at middle; four or five ORBs and MA diverging from R; MA dichotomously branched at middle; MP initially branched near wing base, with both main branches dichotomously branched; CuA distally pectinately branched, CuP dichotomously branched from midlength; a short, oblique mp-cua crossvein present; A1 dichotomously branched; A2 short, but pectinately branched; A3 bifurcated; two gradate series of crossveins proximad outer gradate series. Hindwing much narrower than forewing, ovoid, but strongly narrowed proximad; trichosores present along margin of distal half; a median nygma present between Rs and MA; Sc and R fused distally; single Rs + MA separating from R near wing base; base of MA oblique and slightly sinuate; MP probably dichotomously branched; CuA and CuP both distally pectinately branched; A1 straight with a marginal fork; A2 and A3 simple.

### Etymology

The generic epithet is a combination of *Fiap* (FIAP = The Inter-Asian Philatelic Federation, which has long supported the author WZ’s work on philately) and neural (plural form of the Ancient Greek noun neuron, nerve, vein, and a traditional ending of Neuroptera-like genera). Gender feminine.

### Remarks

The new genus cannot be assigned to any of the families of Psychopsoidea based on our current knowledge of this group (also see the result of phylogenetic analysis). *Fiaponeura* gen. nov. shares the haustellate mouthparts and the presence of more than two forewing ORBs with two closely related families Kalligrammatidae and Aetheogrammatidae (see diagnoses of these families in Yang *et al.*^8^). However, *Fiaponeura* gen. nov. differs from all genera of the latter two families by the much smaller body-size, the presence of nygmata and trichosores, and the sparse crossvenation. Furthermore, the new genus lacks an autapomorphy of Kalligrammatidae, i.e. posterior branch of forewing MP with many distal pectinate branches, consisting of an expansive, triangular region^8^. *Fiaponeura* gen. nov. clearly differs from Psychopsidae and Osmylopsychopidae by the narrowed basal part of forewing costal space with short simple recurrent veinlet, the presence of 4 or 5 forewing ORBs, and of course the haustellate mouthparts. It also lacks a putative autapomorphy of Osmylopsychopidae, i.e. the forewing MA occupying a large area and deeply profusely branched^14^. In Psychopsidae and Osmylopsychopidae, the forewing costal space is broadened with branched recurrent veinlet, while it is continuously broad toward apex in the former family it is strongly narrowed distad in the latter^13^. The mouthparts of all Mesozoic psychopsids and osmylopsychopids are unknown, but they are typically mandibulate in all modern silky lacewings. Notably, *Fiaponeura* gen. nov. and Psychopsidae share some diagnostic forewing features: the presence of only basal nygma, the crossveins in the radial space usually arranged in two or three series, and the MA sparsely branched near midlength. Oswald considered the proliferation of sc-r crossveins and the rather broad costal space in the pterostigmatic area to the autapomorphies of Psychopsidae although the former character state was noted to be possibly homoplasious, to which we also agree^15^. In *Fiaponeura* gen. nov. the costal space is not strongly narrowed as in Osmylopsychopidae, but it is also not as broad as in Psychopsidae.

***Fiaponeura penghiani*****sp. nov.** (Figure 1)

**Diagnosis.** Same as for the genus.

### Description

Body length 7.86 mm; forewing length 11.59 mm, hindwing length 10.95 mm. Head length 0.30 mm, maximum width between outer margins of compound eyes 1.06 mm. Antenna with preserved part 2.47 mm long. Discernible parts of mouthparts: Labrum 0.42 mm long and 0.089 mm wide; prolonged lobe of maxilla 1.25 mm long and 0.077 mm wide; maxillary palp 1.57 mm long and 0.045 mm wide; ligula 0.820 mm long and 0.130 mm wide; labial palp 1.28 mm long and 0.046 mm wide. Prothorax 0.70 mm long and 0.37 mm wide; meso- and metathorax 0.68 mm long and 0.60 mm wide. Foreleg 7.45 mm long; midleg 8.46 mm long, hindleg 6.50 mm long. Abdomen 4.60 mm long.

Head short, feebly domed and setose dorsad. Mouthparts haustellate; labrum short, medially concaved into pair of pointed lobes; maxilla with slenderly elongate, blade-like lobe (putative galea or galea + lacinia), which bears short setae, maxillary palp 5-segmented and much longer than galea, palpomere 3 nearly equal in length to total length of remaining palpomeres; labium with an elongate, thin, hairless ligula, which has a median canal and is bilobed at apex, labial palp 3-segmented and much longer than ligula, palpomere 2 nearly equal in length to total length of remaining palpomeres, palpomere 3 distally with a ovoid sensory area.

Legs slender, densely setose; single tibial spur present; coxa and trochanter short, femur slightly shorter than tibia; tarsus 5-segmented, with tarsomeres 1–5 gradually shortened; pretarsus with a pair of slender claws and a short arolium bearing paired spinous setae.

Wings transparent, with broad transverse dark stripes, and with long setae along margins and veins; forewing with three arcuate stripes on proximal half (each continuous from costal to posterior margin), and with three broader but irregularly shaped patches and a few small markings on distal half; hindwing with rows of stripes extending from costal margin to Rs or MA on proximal half, and with distal half almost entirely dark. Venation in addition to diagnosis of genus: Forewing: More than 20 costal crossveins; most longitudinal veins with end-twigging; 1^st^ and 2^nd^ ORB bifurcated distally, 3^rd^ ORB bifurcated at midlength, 4^th^ ORB branched initially slightly basad midlength and in dichotomous condition, 5^th^ ORB pectinately branched; at least five crossveins present between R and last ORB; number of crossveins between ORBs, MA, MP, and their branches ranging from one to four; five cua-cup crossveins. Hindwing: 22 costal crossveins; most longitudinal veins with end-twigging; six sc-r crossveins; Rs with eight branches, most of which are forked distally but second branch bifurcated near midlength; MA dichotomously branched near midlength; a gradate series of crossveins present among branches of Rs; no crossvein between MP and Cu.

Abdomen slenderly elongate. Genital segments dorsally with a pair of ovoid sclerites as putative ectoprocts, and ventrally seemingly with flatly valvate sclerites although poorly preserved.

### Type material

Holotype: EMTG BU-001679: amber piece preserving an almost complete adult female of *F. penghiani* (wings partly not preserved), a beetle, and a midge; it is polished in the form of a flattened semi-ellipsoid cabochon, clear and transparent, with length ×width about 30.7 × 17.8 mm, height about 3.7 mm.

### Etymology

The new species is dedicated to Mr. Tay Peng Hian, currently the President of the Fédération Internationale de Philatélie (FIP), who over the years has encouraged author WZ to continue his philately.

### Remarks

The genital segments, which possess a pair of ovoid dorsal sclerites and flatly valvate ventral sclerites resemble a general feature of female genitalia of lacewings^17^, suggesting that the holotype of the new species could be a female. It is noteworthy that a strongly elongated ovipositor is present in Kalligrammatidae^8^. Hence, in light of the otherwise great difference in the female genitalia, the new genus and species cannot be assigned to Kalligrammatidae although they share some distinctive morphological similarities, e.g. haustellate mouthparts and presence of multiple forewing ORBs.

**Figure 2:**
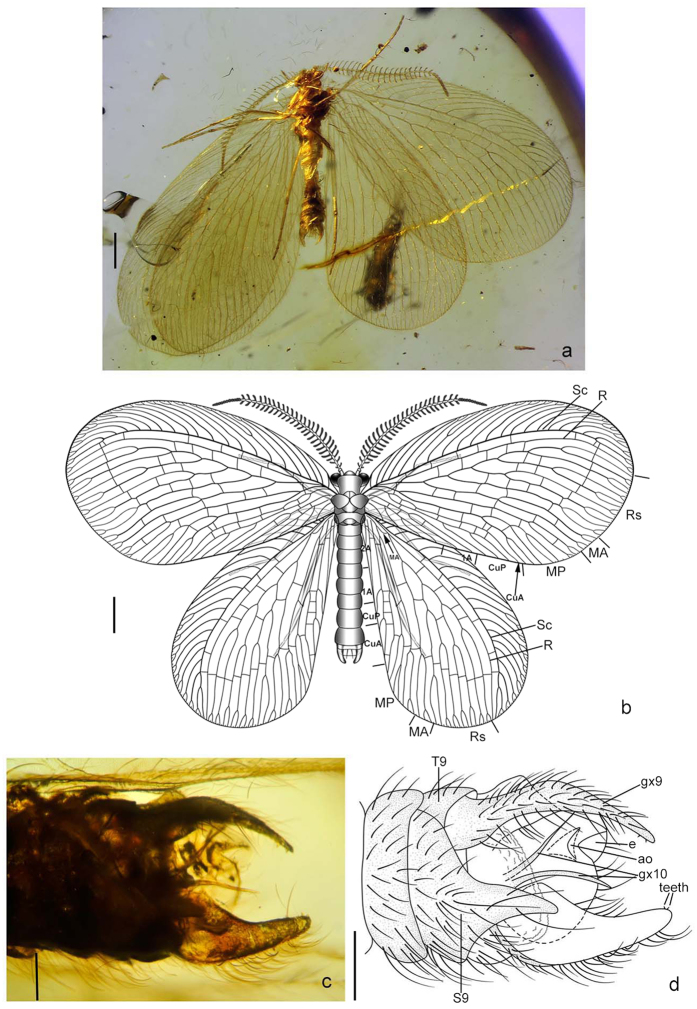
*Cretanallachius magnificu*s Huang *et al*. (**a**) habitus photo, ventral; (**b**) reconstructed habitus drawing; (**c**) photo of male genital segments, ventral; (**d**) drawing of male genital segments, ventral. T: terg S: sternum; e ectoproct; gx: gonocoxite; ao: anal opening. Scale bar: 1 mm (**a,b**); 0.2 mm (**c,d**).

**Genus** ***Cretanallachius*****Huang** ***et al***.^18^ (Figure 2)

**Type species:** *Cretanallachius magnificus* Huang *et al.*^18^: 275, by monotypy.

### Revised diagnosis

Small-sized lacewings (forewing length ~7.70 mm). Head (Fig. 2) orthognathous, feebly domed dorsally. Compound eye with maximum diameter slightly shorter than distance between inner margins of eyes. Ocelli absent. Antenna nearly half as long as forewing, densely hairy, with most flagellomeres bipectinate except for distal 10–12 unbranched flagellomeres. Mouthparts composed of a short, medially slightly concaved labrum, reduced mandibles, and conspicuously elongated maxillae and labium; maxilla with long blade-like lobe (putative galea or galea +lacinia), which is ~3/4 as long as maxillary palp, with truncate tip; labium with paired, elongated, distally pointed ligula, which is ~3/4 as long as labial palp. Pronotum nearly as long as wide. Legs slender, tibial spur absent. Wings broad, moderately veined, immaculate. Forewing ovoid, distinctly broadened distad; trichosores present along distal half of wing margin; nygma absent; costal space rather broad except for narrow base, with a short simple humeral veinlet slightly bent to wing base, and with most costal crossveins distally forked except for some simple ones near wing base, interlink veinlet absent; subcostal space ~1/5 as wide as costal space at middle; distal parts of Sc and R distinctly curved posteriad but not fused with each other; a single Rs +MA diverging from R; Rs with five main branches, each of which bears secondary forking and end-twigging; MA bifurcated at middle; MP initially branched near wing base, anterior branch bifurcated nearly at distal 1/3, posterior branch dichotomously branched from midlength; CuA simple or only with end-twigging, CuP trifurcated from midlength; A1 deeply bifurcated; A2 trifurcated; A3 simple; only one gradate series of crossveins proximad outer gradate series. Hindwing slightly narrower than forewing, ovoid, but strongly narrowed proximad; venation primarily similar to that of forewing; base of MA oblique and slightly sinuate; CuP simple; A1 and A2 present, but short and simple.

### Remarks

*Cretanallachius* was originally described as the first record of the family Dilaridae (pleasing lacewings) in the Mesozoic by Huang *et al.*^18^. Their arguments of its dilarid affinity are the configuration of the similarly shaped fore- and hindwings, the forelegs not raptorial, the prothorax not markedly elongated, the pectinate male antennae, the absence of ocelli, and the veins Sc and R not fused distally. In fact, Dilaridae are characterized by the unipectinate male antennae, the presence of three ocelli-like tubercles on head, the presence of nygmata, the forewing Sc terminating anteriad to wing apex but not curved posteriad, and the elongation of ovipositor^19^. *Cretanallachius* and other dilarids only share the pectinate male antennae, and the other arguments for placing *Cretanallachius* into Dilaridae are unjustified because they are apparently plesiomorphic or homoplasious among lacewing families. However, it is notable that the male antennae of *Cretanallachius* are bipectinate, while in other dilarids the male antennae are unipectinate. This great difference might represent a convergent evolution of the male antennae. More importantly, the male genitalia of *Cretanallachius*, which was erroneously interpreted by Huang *et al.*^18^, fundamentally differs from that in Dilaridae. In Dilaridae the male ectoprocts are a pair of sclerites with a rosette of trichobothria (Nallachinae) or specialized as an internal supraanale (Dilarinae), and the male gonocoxites 9 together with gonocoxites 10 and 11 form an internal complex^17^. The external valvate sclerite of *Cretanallachius* was misinterpreted as the ectoproct^18^ but probably represents the male gonocoxite 9 because it lacks the rosette of trichobothria and also because a true ectoproct (an unpaired dorsal sclerite) is found in our material. Conversely, the broad forewing with posteriorly curved Sc and the haustellate mouthparts suggest the psychopsoid affinity of *Cretanallachius*. Herein, we transfer *Cretanallachius* from Dilaridae to Psychopsoidea, but its familial placement is still uncertain (see the result of phylogenetic analysis).

***Cretanallachius magnificus*** **Huang*****et al**.*^18^ (Figure 2)

*Cretanallachius magnificus* Huang *et al.*^18^: 275.

**Diagnosis.** Same as for the genus.

### Additional description

Body length 4.05–5.02 mm; forewing length 6.47–7.70 mm, hindwing length 5.98–7.70 mm. Head length 0.34–0.50 mm, maximum width between outer margins of compound eyes 0.6–0.9 mm. Antenna with preserved part 3.90 mm long. Discernible parts of mouthparts: Labrum 1.80 mm long and 0.045 mm wide; prolonged lobe of maxilla 0.36 mm long and 0.014 mm wide; maxillary palp 0.76 mm long and 0.027 mm wide; ligula 0.52 mm long and 0.010 mm wide; labial palp 0.70 mm long and 0.045 mm wide. Prothorax 0.21 long and 0.52 mm wide; meso- and metathorax 0.66 mm long and 1.02 mm wide. Foreleg 3.82 mm long; midleg 3.96 mm long, hindleg 4.29 mm long. Abdomen 3.80 mm long.

Mouthparts haustellate; labrum short, medially slightly concaved; mandibles absent; maxilla with slenderly elongate, glabrous, blade-like lobe (putative galea or galea +lacinia), maxillary palp 5-segmented, much longer than galea, palpomere 5 slightly longer than each of other palpomeres; labium with a pair of elongate, thin, glabrous, and distally pointed ligula, labial palp 3-segmented, much longer than ligula, palpomere 2 nearly equal in length to total length of remaining palpomeres.

Male genitalia covered with long setae; an unpaired, flat, broad sclerite (putative ectoproct) present dorsally, it is concaved medially and distally associated with trumpet-shaped opening of anus on ventral side; sternum 9 strongly projecting medially into a horn-like process; a pair of large valvate sclerites (putative gonocoxites 9) present laterally, narrowed distad with two or three small inner teeth; an internal structure present, composed of a broad membranous, rugous sac, and a pair of slenderly elongate and spinous lobes (putative gonocoxites 10).

### Materials examined

PCXJ BA-0001: amber piece preserving a complete adult male of *C. magnificus*, a jumping bristletail, a bark louse, and a true bug; it is polished in the form of a flattened semi-ellipsoid cabochon, clear and transparent, with length ×width about 26.3 × 18.0 mm, height about 7.3 mm. PCXJ BA-0002: amber piece preserving an adult male of *C. magnificus* (wings partly not preserved); it is polished in the form of a flattened semi-ellipsoid cabochon, clear and transparent, with length × width about 11.8 × 9.5 mm, height about 5.1 mm. PCXJ BA-0003: amber piece preserving a complete adult male of *C. magnificus* and a beetle; it is polished in the form of a flattened semi-ellipsoid cabochon, clear and transparent, with length ×width about 26.4 × 20.0 mm, height about 6.1 mm.

**Figure 3:**
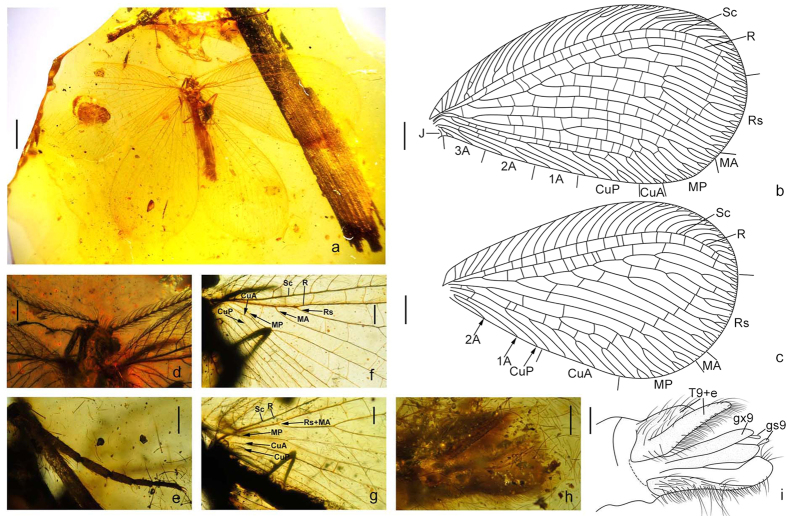
*Burmopsychops limoae* gen. et sp. nov. (**a**) habitus photo, dorsal; (**b**) forewing venation; (**c**) hindwing venation; (**d**) photo of head, dorsal; (**e**) photo of hindleg tarsus; (**f**) photo of proximal part of forewing; (**g**) photo of proximal part of hindwing; (**h**) photo of female genital segments, ventral; (**i**) drawing of female genital segments, ventral. T: tergum; e: ectoproct; gx: gonocoxite; gs: gonostylus. Scale bar: 2 mm (**a**); 1 mm (**b,c**); 0.5 mm (**d,f,g**); 0.05 mm (**e**); 0.2 mm (**h,i**).

**Figure 4:**
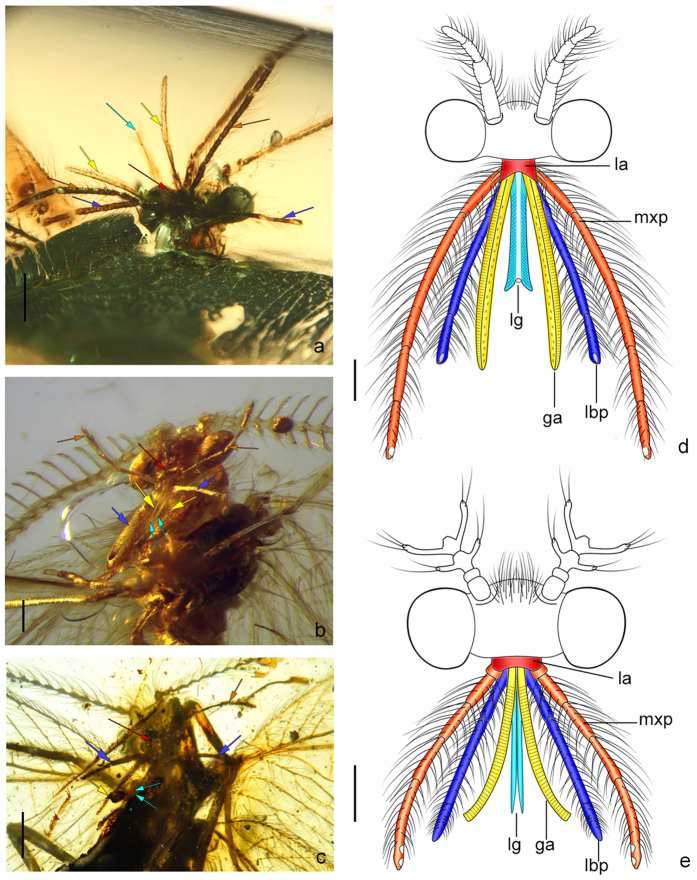
Mouthparts of Burmese long-proboscid lacewings with homology assessment of elongated elements. (**a**) *Fiaponeura penghiani* gen. et sp. nov.; (**b**) *Cretanallachius magnificus* Huang *et al.*; (**c**) *Burmopsychops limoae* gen. et sp. nov.; (**d**) reconstruction of mouthparts of *F. penghiani* gen. et sp. nov.; (**e**) reconstruction of mouthparts of *C. magnificus*. la: labrum; ga: galea; mxp: maxillary palp; lg: ligula; lbp: labial palp. Color of arrows corresponds to color of reconstructed mouthparts. Scale bar: 0.5 mm (**a,c**); 0.2 mm (**b,d,e**).

**Genus** ***Burmopsychops*** **gen. nov.** (Figures 3 and 4)

**Type species:** *Burmopsychops limoae* sp. nov.

### Diagnosis

Small-sized lacewings (forewing length ~7.7 mm). Head (Figs 3 and 4) orthognathous, feebly domed dorsally. Compound eye with maximum diameter slightly shorter than distance between inner margins of eyes. Ocelli absent. Antenna moniliform, nearly half length of forewing, densely hairy. Mouthparts composed of a short, medially slightly concaved labrum, reduced mandibles, and conspicuously elongated maxillae and labium; maxilla with long blade-like lobe (putative galea or galea +lacinia), which is ~3/4 as long as maxillary palp with truncate tip; labium with paired, elongated, distally pointed ligula, which is ~3/4 as long as labial palp. Pronotum nearly as long as wide. Legs slender, tibial spur absent. Wings broad, moderately veined, immaculate. Forewing ovoid, distinctly broadened distad; trichosores present along distal half of wing margin; nygma absent; costal space rather broad except for narrow base, with a short simple humeral veinlet slightly bent to wing base, and with many costal crossveins distally forked except for some simple ones on proximal half; subcostal space ~1/5 as wide as costal space at middle; distal parts of Sc and R distinctly curved posteriad but not fused with each other; Rs with seven main branches, each of which bears secondary forking and end-twigging, except anterior two branches only with end-twigging; MA diverging from R and bifurcated distad; MP initially branched near wing base, anterior branch bifurcated nearly at distal 1/4, posterior branch dichotomously branched from midlength; CuA trifurcated distad, CuP pectinately branched from midlength; A1 distally bifurcated; A2 and A3 deeply bifurcated near wing base; J short and simple; a number of crossveins present proximad outer gradate series. Hindwing slightly narrower than forewing, ovoid, but strongly narrowed proximad; venation primarily similar to that of forewing; single Rs + MA separating from R near wing base; CuP probably deeply dichotomously branched; CuA pectinately branched; CuP simple; A1 and A2 present, but simple.

### Etymology

The generic epithet is a combination of *Burma* (Myanmar) and *psychops* (a common ending of Psychopsoidea). Gender masculine.

### Remarks

The new genus resembles *Cretanallachius* by having similar mouthpart features and wing venation, but can be separated from the latter genus based on the forewing MA separating from R, the distally bifurcated forewing CuA, and the absence of hindwing MA base. In *Cretanallachius* the forewing MA is diverged from Rs, the forewing CuA is simple, and a sinuate hindwing MA base is present. It is notable that the antenna of *Burmopsychops* gen. nov. is not bipectinate as that in *Cretanallachius*. However, the antenna of *Cretanallachius* might be sexually dimorphic although the female of this genus is still unknown, while *Burmopsychops* gen. nov. is only known from the female. Therefore, we cannot use this antennal feature to distinguish these two genera at present.

***Burmopsychops limoae*** **sp. nov.** (Figures 3 and 4)

**Diagnosis.** Same as for the genus.

### Description

Body length 7.33 mm; forewing length 11.36 mm, hindwing length 10.20 mm. Head length 0.29 mm, maximum width between outer margins of compound eyes 0.88 mm. Antenna with preserved part 4.92 mm long. Discernible parts of mouthparts: Labrum 0.26 mm long and 0.070 mm wide; prolonged lobe of maxilla 0.94 mm long and 0.066 mm wide; maxillary palp 1.26 mm long and 0.017 mm wide; ligula 0.74 mm long and 0.0064 mm wide; labial palp 1.13 mm long and 0.039 mm wide. Prothorax 0.47 long and 0.45 mm wide; meso- and metathorax 1.16 mm long and 0.94 mm wide. Foreleg 3.43 mm long; midleg 4.30 mm long, hindleg 3.70 mm long. Abdomen 5.62 mm long.

Head short, feebly domed and setose dorsad. Mouthparts haustellate; labrum short, medially concaved into pair of rounded lobes; maxilla with slenderly elongate, blade-like lobe (putative galea or galea + lacinia), which bears short setae, maxillary palp 5-segmented, much longer than galea, palpomeres gradually lengthened; labium with a pair of elongate, thin, hairless ligula, labial palp 3-segmented, much longer than ligula, palpomere 2 nearly equal in length to total length of remaining palpomeres, palpomere 3 distally with an ovoid sensory area.

Legs slender, densely setose; single tibial spur present; coxa and trochanter short, femur slightly shorter than tibia; tarsus 5-segmented, with tarsomeres 1–5 gradually shortened; pretarsus with a pair of slender claws and a short arolium bearing paired spinous setae.

Wings transparent, with long setae along margins and veins; Venation in addition to diagnosis of genus: Forewing: More than 20 costal crossveins; half of the longitudinal veins with end-twigging; Rs with seven main branches, 1^st^ and 6^th^ branches simple with end-twigging, 2^nd^ through 5^th^ branched in the midlength, with end-twigging; at least fourteen crossveins present between R and Rs; number of crossveins between Rs, MA, MP, and their branches ranging from one to eleven; twelve cua-cup crossveins. Hindwing: 28 costal crossveins; distal longitudinal veins with end-twigging; thirteen sc-r crossveins; Rs with six branches, posterior two branches forked distally but remaining branches bifurcated near midlength; MA dichotomously branched near midlength, but base of MA absent; a gradate series of crossveins present among branches of Rs and MA; seven crossveins between MP and CuA.

Abdomen slenderly elongate. Female genitalia covered with long setae; a pair of flat, broad sclerites (putative fused tergum 9 and ectoprocts) present dorsally; a pair of prolonged sclerites (putative gonocoxites 9), which distally bear a pair of digitiform processes (putative gonostyli 9), present ventrally as ovipositor.

### Type material

Holotype: EMTG BU-001293: amber piece preserving a complete adult female of *B. limoae*, three cockroaches, and a lacewing larva; it is polished in the form of a flattened trapezoidal cabochon, clear and transparent, with length × width about 34.8 × 31.4 mm, height about 4.5 mm.

### Etymology

The new species is dedicated to Mrs. Mo Li, who kindly donated the piece of amber that includes the new species.

### Phylogenetic analysis

**Figure 5:**
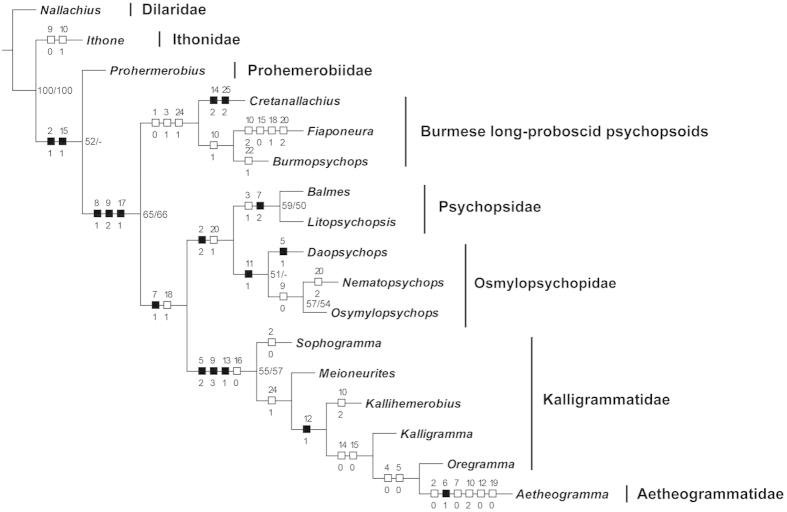
A phylogeny of Psychopsoidea. Topology represents the most parsimonious tree yielded from NONA. Unambiguous morphological character state changes are shown on the tree. Bootstrapping/jackknife support values are shown at relevant nodes.

The parsimony analysis yielded a single most parsimonious tree (MPT) (length = 67, consistency index = 50, retention index = 65) as shown in Fig. 5. All genera of Psychopsoidea are assigned into a monophyletic group based on the distinctly posteriorly curved forewing Sc or Sc + R (char. 8:1), the presence of numerous forewing sc-r crossveins (char. 9:2), and the broadly subtriangular forewing (char. 17:1). The three long-proboscid genera from the mid-Cretaceous of Myanmar form a monophyletic group, which is supported by the absence of branched recurrent forewing humeral veinlet (char. 1:0), the forewing costal space not narrowed distad (char. 3:1), and the presence of long proboscis (char. 24:1), and it is the sister group of the clade comprising the remaining genera representing Aetheogrammatidae, Kalligrammatidae, Osmylopsychopidae and Psychopsidae. The sister group relationship between *Fiaponeura* gen. nov. and *Burmopsychops* gen. nov. is supported by the presence of two or more longitudinal veins (i.e. MA and Rs) separated from R (char. 10:1). However, all aforementioned character states are homoplasious, and the nodal supports are very low for the relationships among the three Burmese long-proboscid genera. Within the clade comprising the remaining genera, Osmylopsychopidae (represented by *Daopsychops*, *Nematopsychops*, and *Osmylopsychops*) and Psychopsidae (represented by *Balmes* and *Litopsychopsis*) are assigned to be sister group, supported by the proximally distinctly broadened forewing costal space (char. 2:2) and the presence of nygmata (char. 20:1), corroborating their close relationship proposed by Peng *et al.*^13^. Two character states, i.e. the forewing costal space not narrowed distad (char. 3:1) and the specialized configuration of forewing Sc and R (char. 7:2), support the monophyly of Psychopsidae. These two characters states are among the proposed synapomorphies of the whole Psychopsidae^13^. The deeply profusely branched forewing MA (char. 11:1) is assigned as an autapomorphy of Osmylopsychopidae. Considering Aetheogrammatidae and Kalligrammatidae, the genera of these two families form a monophyletic group supported by four character states, i.e., the presence of interlinked veinlets between crossveins of whole forewing costal space (char. 5:2), the presence of more than 30 forewing sc-r crossveins (char. 9:3), the extremely dense crossvenation (char. 13:1), and the absence of forewing outer gradate series of crossveins (char. 16:0). However, the monophyly of Kalligrammatidae is not recovered due to the position of aetheogrammatid genus *Aetheogramma* assigned as the sister group of the kalligrammatid genus *Oregramma*. This result is consistent with the previous hypothesis that Aetheogrammatidae might be a specialized branch of Kalligrammatidae^20^.

It should be noted that our phylogenetic analysis was not intended to address the phylogeny of Psychopsoidea with present limited sampling but to assess the positions of the three long-proboscid genera. The systematics of Mesozoic psychopsoids is complicated and flooded with many taxa with unclear taxonomic and phylogenetic status^13^. Moreover, the unambiguous autapomorphy of Psychopsoidea has yet not been found. The three character states herein assigned to support the monophyly of Psychopsoidea can be also found in some species of other lacewing families, e.g. Ithonidae^21^,^22^. Actually, besides the psychopsoid families, the three Burmese long-proboscid lacewing genera only resemble some Tertiary ithonids previously placed in Polystoechotidae^22^ by the broadly subtriangular forewing with dense venations and posteriorly curved Sc and R. However, they greatly differs from ithonids by the lacking of branched recurrent humeral veinlet and by the posterior branch of forewing MP not pectinately branched. As such, the placement of these Burmese long-proboscid genera in Psychopsoidea is reasonable, although they cannot be placed into any of the known psychopsoid families. Admittedly, it is to some extent arbitrary to establish a new family name for these genera due to lack of a convincing autapomorphy, although all of them possess unique configuration of long-proboscid mouthparts and some antennal or wing features. Knowledge of larval morphology and genital features would be essential to further reveal the familial affinities of these enigmatic genera.

## Discussion

**Figure 6:**
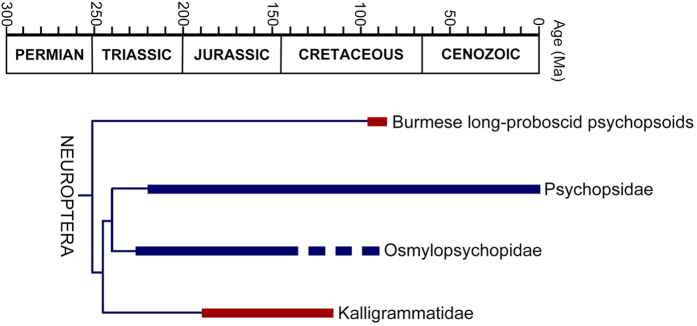
Evolutionary chronogram of Psychopsoidea. Interfamilial phylogeny based on present results, thick lines indicate known geological distributions. Lineages with long-proboscid mouthparts highlighted red.

In light of the present newly described fossils, long-proboscid lacewings consist of at least four independent lineages, i.e. Permithonidae, Kalligrammatidae + Aetheogrammatidae, *Fiaponeura* gen. nov., and *Cretanallachius* + *Burmopsychops* gen. nov. (Fig. 6). Permithonidae represent the oldest known long-proboscid lacewings, but only one species (i.e., *Tschekardithonopsis? oblivius* Vilesov)^23^ from the Lower Permian (Kungurian) is confirmed to have siphonate mouthparts^12^. Its proboscis, which is considered to be conjoined by maxillary palpal elements, is nearly double the length (1.7 mm long) of the head; furthermore there is a narrow median food canal and an apical setose region^12^. The mouthparts of Kalligrammatidae are documented to a greater degree; most kalligrammatids have siphonate mouthparts except for species of Sophogrammatinae^8^,^12^,^24^. The proboscides of kalligrammatids are variable in length (11–25 mm long) among species^8^,^12^. Nonetheless, one basic feature of the kalligrammatid siphonate mouthparts seems to be constant, namely the absence of labial palps^24^. It was reported that the kalligrammatid proboscis consists of conjoined maxillary galeae to form a tubular siphon that is anatomically similar to that of the lepidopteran Glossata^12^. However, a siphon with two largely separate elongated parts is found in an undescribed kalligrammatid from China (see Labandeira & Currano: Fig. 6f 24), possibly indicating a more complex structural feature of the kalligrammatid proboscis.

The elongated elements of the mouthparts in the amber psychopsoids described herein undoubtedly belong to maxillae and/or labium. However, due to the highly specialized condition, it is a little bit difficult to accurately interpret the homology of these prolonged structures. In *Fiaponeura* gen. nov., the paired long lobes are very likely the parts of maxillae, while the central long and distally bifid lobe is probably the ligula of the labium, as having similar groundplan to the typical neuropteran ligula. The elongated lobe of maxilla in *Fiaponeura* gen. nov. could be galea with reduction of lacinia or represent a structure fused by galea and lacinia (such fusion might be inferred by the longitudinal line within this structure as shown in Fig. 4d, but this could be also a secondary character). Nevertheless, as the complete fusion of galea and lacinia is rarely found in Neuroptera, it is more likely that this long lobe is just a specialized galea, and the lacinia is lost in relation to the reduction of other mouthparts’ elements, such as the mandibles.

In *Cretanallachius* and *Burmopsychops* gen. nov., the lateral pair of long lobes is probably homologous with that in *Fiaponeura* gen. nov., being as a part of maxillae. However, attribution of the other pair of long lobes positioned centrally is unclear. As the ligula is rarely separated in most lacewings, these lobes might have to be interpreted as specialized lacinia. In this case, the ligula should have been completely lost in *Cretanallachius* and *Burmopsychops* gen. nov. Nonetheless, based on the central position of these lobes, they may be homologous with the central lobe in their contemporary and phylogenetically related genus *Fiaponeura* gen. nov. as a part of labium (putative ligula), which we favored at present (Fig. 4e).

Both maxillary and labial palps are well developed and much longer than the two pairs of long lobes in these Burmese amber psychopsoids, while only one pair of palps, putatively the maxillary palps, are retained in kalligrammatids^24^. The tubular siphon as in kalligrammatids is also not found in these species. Instead, the prolonged elements of mouthparts in the material we examined are distinctly separate distally. In *Fiaponeura* gen. nov. the long blade-like lobe is slightly curved along the outer margin; it is equipped with a longitudinal ridge (a dark line medially) and some cuticular structures, such as transverse microgrooves and coarse setae. Such features imply that the lobes could temporarily come together, possibly enclosing the ligula, and thus form a functional proboscis. The ligula of *Fiaponeura* gen. nov. bears a conspicuous tubular food canal in the middle, numerous oblique microgrooves that line the food canal, and a pair of acutely pointed terminal lobes. Comparably, the elongated lobes in *Cretanallachius* and *Burmopsychops* gen. nov. are gracile and bear much simpler cuticular structures with only transverse microgrooves on the lateral lobes (specialized galeae or galeae +laciniae), which might similarly function as those in *Fiaponeura* gen. nov. to form a temporary proboscis.

Multiple lineages of Middle Mesozoic insects with siphonate proboscides were reported to be responsible for pollination, such as several lineages of true flies^25^,^26^, three lineages of scorpionflies belonging to the Aneuretopsychina clade^27^, and the lacewings belonging to the nonsophogrammatine Kalligrammatidae^24^. Plants that may have been pollinated by these long-proboscid insects include corystospermalean and caytonialean seed ferns, ginkgophytes, cheirolepidiaceous conifers, gnetaleans, and bennettitaleans^24^. These plants usually show morphological modifications of their ovulate organs, e.g., the micropyle is significantly lengthened or hidden deep in a cupulate structure^12^. Inference of the long-proboscid pollination mode in the mid-Mesozoic is challenging due to lack of direct evidence in the association between host plants and pollinators in the fossil record. However, Labandeira outlined several categories of useful evidence for establishment of pollinator mutualisms in fossil records: 1) the structure of insect mouthparts and related head features; 2) the structure of ovulate organs and especially features for provisioning rewards for insects; 3) the structure of pollen and its placement on plant and insect contact surfaces; 4) palynivore gut contents and dispersed coprolites; 5) the host occurrence, geometry, and feeding patterns of insect plant damage on reproductive structures; and 6) knowledge of the present-day life histories of the nearest, closest relatives of both fossil plant hosts and their insect pollinators^12^. Application of the above evidence uncovered Middle Mesozoic pollinator mutualisms in thrips, scorpionflies, and true flies^26^,^27^,^28^.

In the long-proboscid psychopsoids herein reported, we were unable to identify the presence of pollen grains attached to any specimen. Furthermore, we lack data on chemical compounds from mouthparts and digestive system for diet estimation. However, based on known life histories of modern lacewings, as well as the pollination habit of long-proboscid kalligrammatids, these long-proboscid amber lacewings are more likely to be pollinators feeding on nectar and possibly pollen, rather than hematophages or piercing-sucking herbivores. Remarkably, the proboscides of these amber lacewings are definitely among the shortest of mid-Mesozoic insect pollinators with haustellate or siphonate mouthparts. The temporarily formed proboscides of *Fiaponeura* gen. nov. and *Burmopsychops* gen. nov. are ~1.0 mm in length, while in *Cretanallachius* the proboscis is considerably shorter (~0.4 mm). In previous studies, candidate host plants for mid-Mesozoic pollinator insects with proboscides ranging from 0.9 to 1.8 mm in length were inferred to be Pentoxylaceae, which have a diminutive micropyle and salpinx tube (2 mm maximum length by 0.1 mm diameter), and lineages of Bennettitales with surface accessible ovules^25^. Conversely, the distance and width of the conduit from the aperture entry to the pollen chamber was much greater in other plant lineages reported in mid-Mesozoic pollination mutualisms, such as Caytoniaceae, Cheirolepidiaceae, Czekanowskiaceae, and Gnetales^27^. Therefore, it is reasonable to hypothesize that a pollination mutualism existed between the small-sized long-proboscid lacewings from the mid-Cretaceous of Myanmar and the above mentioned co-occurring plants (i.e. Pentoxylaceae and Bennettitales) with relatively shallow-seated pollen-receptive areas. However, it is noteworthy that angiosperms were also present in mid-Cretaceous of Myanmar, and different types of angiosperm flowers, some of which are small-sized, are known in this deposit though they are still poorly identified^24^,^29^,^30^. As such, we cannot eliminate the possibility that these Burmese amber psychopsoids with small-sized proboscides were pollinators of certain angiosperm plants. However, previous studies indicate that the earliest angiosperm pollination was predominantly associated with small nonproboscid, large mandibulate, and punch-and-sucking insects^31^,^32^ and the earliest deep-throated flowers specialized for long-proboscid insect pollination are of Turonian age (ca. 45 million years after the earliest angiosperm fossils)^33^. Furthermore, the extinction of these long proboscid psychopsoids is more difficult to be explained if they were associated with angiosperms that was rising since mid-Cretaceous.

The origin and diversification of Neuroptera, which is estimated to have occurred no later than the Late Permian^34^, is the most spectacular event in the evolution of Neuropterida. Based on a time-scale of lacewing diversification estimated by Wang *et al.*^35^, 10 of the 16 extant neuropteran families were present by the end of the Triassic, while the divergence of the remaining modern families occurred no later than the Early Cretaceous. It is remarkable that the number of neuropteran families approximately doubled from the Early Jurassic to the end of the Early Cretaceous, although half of them became extinct by the end of the Late Cretaceous^36^. The Triassic diversification of Neuroptera, which included innovations of remarkable biological traits in the larvae, such as the subterranean phytophagous Ithonidae and debris-carrying Chrysopidae and Myrmeleontiformia, is considered to be correlated to a floral change from archaic lycopsids, ferns, cordaites and pteridosperms to radiations of cycads, ginkgos, conifers and angiosperm-like Bennettitales in the Triassic^35^. Various insect-plant associations involving Neuroptera, e.g., leaf mimesis in Saucrosmylidae^35^, debris-carrying mimesis in Chrysopoidea^37^, and gymnosperm pollinating Kalligrammatidae^12^, are vivid examples of the ‘golden age’ of Neuroptera in the mid-Mesozoic when the ancient flora was dominated by gymnosperms, which subsequently underwent a dramatic decline with the rise of angiosperms in the Late Cretaceous. The new long-proboscid lacewings from mid-Cretaceous of Myanmar serve as an exceptional example for understanding of the long-proboscid pollination mode during the significant floral change in the Cretaceous and highlight the early evolution of lacewings.

## Methods

The Burmese ambers investigated here originated from the Hukawng Valley in Tanaing Township, Myitkyina District of Kachin State, Myanmar. The age of this deposit has been dated at 98.8 ± 0.6 million years using U-Pb dating of zircons from the volcaniclastic matrix of the amber^38^. All investigated specimens are currently housed in the Entomological Museum, China Agricultural University (CAU), Beijing. Type specimens will eventually be deposited in the Three Gorges Entomological Museum (EMTG), Chongqing (specimens available for study by contacting XL or WZ), while the remaining specimens all attributed to one described species, *Cretanallachius magnificus* Huang *et al.*^18^, will eventually be deposited in the personal collection of Xiao Jia (PCXJ), Kunming (specimens available for study by contacting XL or WZ).

Photographs and drawings were prepared using a Zeiss SteREO Discovery V12 stereo microscope system, a DM 2000 Leica microscope, and a Nikon D800 digital camera. Measurements were taken under a Keyence VHX-1000 microscope, equipped with a VH-Z20R lens. Morphological terminology of adult generally follows Aspöck *et al.* for the wing venation^19^ and Aspöck & Aspöck for the genitalia^17^. Abbreviations of wing venation are as follow: A, anal vein; Cu, cubitus; CuA, cubitus anterior; CuP, cubitus posterior; MA, media anterior; MP, media posterior; ORB, oblique radial branch; R, radius; Rs, radial sector; Sc, subcosta.

To test the phylogenetic status of the psychopsoid genera herein described, we performed a phylogenetic analysis by sampling 14 ingroup taxa, including *Burmopsychops* gen. nov., *Cretanallachius*, *Fiaponeura* gen. nov., and 11 other genera representing all four families of Psychopsoidea (see Supplementary Materials). We selected *Nallachius* Navás, 1909 (Dilaridae), *Ithone* Newman, 1838 (Ithonidae), and *Prohemerobius* Handlirsch, 1907 (Prohemerobiidae) as the outgroup taxa because they represent relatively basal lineages of Psychopsoidea^5^,^36^. Specimens of all extant taxa were examined by the authors, and the character states of fossil taxa belonging to Aetheogrammatidae, Kalligrammatidae, Osmylopsychopidae and Psychopsidae were determined based reliable and detailed illustrations in the literature^8^,^13^,^39^,^40^.

The morphological characters used in the phylogenetic analysis comprise 25 adult characters (see Supplementary Materials). Unknown characters were coded as “?”. The data matrix is given in Supplementary Data.

The analysis was performed using WinClada ver. 1.00.08^41^ and NONA ver. 2.0^42^. The heuristic search was used with maximum trees to keep setting to 10000 and number of replication setting to 100. The bootstrap branch support values were calculated in NONA ver. 2.0. All characters were treated as unordered and with equal weight. Character states were mapped on the strict consensus tree, which shows only unambiguous changes.

## Additional Information

**How to cite this article**: Lu, X.-M. *et al.* New long-proboscid lacewings of the mid-Cretaceous provide insights into ancient plant-pollinator interactions. *Sci. Rep.* **6**, 25382; doi: 10.1038/srep25382 (2016).

## Supplementary Material

Supplementary Information
