# Supplementary material for: New long-proboscid lacewings of the mid-Cretaceous provide insights into ancient plant-pollinator interactions
Source: Sci Rep. 2016 May 5;6:25382. doi: 10.1038/srep25382 (PMC4857652; doi:10.1038/srep25382)
Supplement: Supplementary Information [file srep25382-s1.pdf]

Supplementary Materials.

New long-proboscid lacewings of the mid-Cretaceous provide insights into ancient plant-pollinator interactions

Xiu-Mei Lu[a], Wei-Wei Zhang[b], Xing-Yue Liu[a]*

[a]Department of Entomology, China Agricultural University, Beijing 100193, China; [b]Three Gorges Entomological Museum, P.O. Box 4680, Chongqing 400015, China.

*Corresponding authors: Xingyue Liu xingyue_liu@yahoo.com

Supplementary Table S1. Measurements of mouthpart elements in the studied lacewing species (mm).

**Taxa**	**labrum**		**galea**		**maxillary palp**		**ligula**		**labial palp**	
	length	width	length	width	length	width	length	width	length	width
***Fiaponeura penghiani* gen. et sp. nov.**	0.42	0.089	1.25	0.077	1.57	0.045	0.820	0.006	1.28	0.046
***Cretanallachius magnificus* Huang et al.**	1.80	0.045	0.36	0.014	0.76	0.027	0.52	0.010	0.70	0.045
***Burmopsychops limoae* gen. et sp. nov.**	0.260	0.070	0.940	0.066	1.26	0.017	0.740	0.006	1.13	0.039

Supplementary Table S2. Morphological character matrix used in the phylogenetic analysis.

	0	0	0	0	0	0	0	0	0	1	1	1	1	1	1	1	1	1	1	2	2	2	2	2	2
	1	2	3	4	5	6	7	8	9	0	1	2	3	4	5	6	7	8	9	0	1	2	3	4	5
Nallachius	0	0	0	0	0	0	0	0	1	0	0	0	0	0	0	0	0	0	0	1	0	0	0	0	1
Ithone	1	0	0	1	0	0	0	0	0	1	0	0	0	0	0	1	0	0	0	0	0	0	1	0	0
Prohermerobius	1	1	0	1	0	0	0	0	1	0	0	0	0	0/1	1	?	0	0	0	0	?	?	?	?	?
Kallihemerobius	1	1	0	1	2	0	1	1	3	2	0	1	1	1	1	0	1	1	1	0	1	?	1	1	0
Kalligramma	?	1	0	1	2	0	1	1	3	0	0	1	1	0	0	0	1	1	1	0	1	1	1	1	0
Oregramma	?	1	0	0	0	0	1	1	?	0	0	1	1	0	0	0	1	1	1	0	1	1	1	1	0
Sophogramma	1	0	0	1	2	0	1	1	3	0	0	0	1	?	?	0	1	1	0	0	1	1	1	0	0
Meioneurites	?	1	0	1	?	?	?	?	?	?	0	0	1	?	?	0	1	?	?	?	1	?	?	1	?
Balmes	1	2	1	1	0	0	2	1	2	0	0	0	0	1	1	1	1	1	0	2	0	1	1	0	0
Litopsychopsis	?	2	1	1	?	0	2	1	?	?	?	?	0	1	1	1	1	1	0	?	0	?	?	?	?
Daopsychops	1	2	0	1	1	0	1	1	2	0	1	0	0	1	1	1	1	?	0	1	1	?	?	?	?
Nematopsychops	1	2	0	1	0	0	1	1	0	0	1	0	0	1	1	1	1	1	0	2	1	0	?	?	?
Osymylopsychops	1	2	0	1	0	0	1	1	0	0	1	0	0	1	1	?	1	?	0	1	1	?	?	?	?
Fiaponeura	0	1	?	1	0	?	?	1	2	2	0	0	0	1	0	1	1	1	0	2	0	0	1	1	0
Cretanallachius	0	1	1	1	0	0	0	1	2	0	0	0	0	2	1	1	1	0	0	0	0	0	1	1	2
Burmopsychops	0	1	1	1	0	0	0	1	?	1	0	0	0	1	1	1	1	0	0	0	0	1	1	1	0
Aetheogramma	0	0	0	0	0	1	0	1	3	2	0	0	1	0	0	0	1	1	0	0	1	1	1	1	?

Supplementary Text.

List of taxa used in the phylogenetic analysis.

Family Dilaridae Newman, 1853

Genus *Nallachius* Navás, 1909

Family Ithonidae Newman, 1853

Genus *Ithone* Newman, 1838

Family Prohemerobiidae Handlirsch, 1907

Genus *Prohemerobius* Handlirsch, 1907

Family Psychopsidae Handlirsch, 1906

Genus *Balmes* Navás, 1910

Genus *Litopsychopsis* Engel & Grimaldi, 2008

Family Osmylopsychopidae Martynova, 1949

Genus *Daopsychops* Peng et al. 2015

Genus *Nematopsychops* Peng et al. 2015

Genus *Osmylopsychops* Tillyard, 1923

Family Kalligrammatidae Handlirsch, 1906

Genus *Sophogramma* Ren & Guo, 1996

Genus *Meioneurites* Handlirsch, 1906

Genus *Kallihemerobius* Ren & Oswald, 2002

Genus *Kalligramma* Walther, 1904

Genus *Oregramma* Ren, 2003

Family Aetheogrammatidae Ren & Engel, 2008

Genus *Aetheogramma* Ren & Engel, 2008

Family incertae sedis

Genus *Fiaponeura* gen. nov.

Genus *Cretanallachius* Huang et al. 2015

Genus *Burmopsychops* gen. nov.

List of characters for the phylogenetic analysis.

Forewing:

1. Branched recurrent humeral veinlet: (0) absent; (1) present.

2. Ratio of maximum width of costal space/subcostal space: (0) less than 3:1; (1) 4:1-5:1; (2) more than 5:1.

3. Costal space: (0) narrowed distad; (1) not narrowed distad.

4. Costal crossveins: (0) mostly simple; (1) with many forked ones.

5. Interlinked veinlets between costal crossveins: (0) absent; (1) present only on proximal half; (2), present along entire costal space.

6. Sc: (0) distally branched; (1) distally simple.

7. Sc and R: (0) not fused distad; (1) fused distad, forming a sharp angle; (2) fused distad by an abrupt subcostal curve, which is continuous with a short crossvein connecting to Rs.

8. Position of Sc: (0) entering wing margin proximal to or within pterostigmatic area; (1) entering wing margin posteriad pterostigmatic area.

9. Number of sc-r crossveins: (0) 1; (1) 0; (2) less than 20; (3) more than 30.

10. Oblique radial branches (ORB): (0) absent; (1), present, but only one; (2) present, with two or

more ORBs.

11. MA occupying a large area and profusely branched near its base: (0) no; (1) yes.

12. MP occupying a large subtriangular area and pectinately branched near its base: (0) no; (1) yes.

13. Crossveination: (0) modest; (1) extremely dense.

14. CuA: (0) dichotomously branched; (1) pectinately branched; (2) simple.

15. CuP: (0) dichotomously branched; (1) pectinately branched.

16. Outer gradate series of crossveins: (0) absent; (1) present.

17. Wing shape: (0) elongate and elliptical; (1) broad and subtriangular.

18. Wing shape: (0) similarly shaped with hindwing; (1) differently shaped with hindwing.

19. Eyespot: (0) absent; (1) present.

20. Nygma: (0) absent; (1) proximal and median nygmata present; (2) only proximal nygma present.

21. Trichosores: (0) present; (1) absent.

Hindwing:

22. Sinuate oblique MA base: (0) present; (1) absent.

Head:

23. Three ocellus-like tubercles: (0) present; (1) absent.

24. Type of mouthparts: (0) chewing mandibulate; (1) siphonate.

25. Male antenna: (0) filiform; (1) unipectinate; (2) bipectinate.
